# Antioxidant and the Dwarfing Candidate Gene of “Nantongxiaofangshi” (*Diospyros kaki* Thunb.)

**DOI:** 10.1155/2019/1629845

**Published:** 2019-11-25

**Authors:** Yuhan Dong, Peihong Wang, Mengting Jiang, Shenchun Qu

**Affiliations:** ^1^College of Horticulture, Nanjing Agricultural University, Nanjing 210095, China; ^2^Jiangsu Key Laboratory for Horticultural Crop Genetic Improvement, Nanjing 210014, China

## Abstract

The aims of this work were to identify genes related to dwarfing for subsequent dwarfing-related research in persimmon and evaluate the relationship between antioxidant activity, dwarf, and hormones of persimmon trees for analyzing the possible dwarf mechanism oxidation factors. In the present study, a transcriptome analysis of “Nantongxiaofangshi” was used to identify and clone 22 candidate genes related to gibberellin signal transduction pathways and synthetic pathway. The expression of these genes was assessed in two persimmon cultivars, “Dafangshi” and “Nantongxiaofangshi,” by RT-qPCR at different phenological stages and in response to the exogenous application of GA_3_ (GA treatment) and PAZ (paclobutrazol, a plant growth inhibitor, also called PP_333_). The results revealed differential expression of 14 of these 22 genes in the two varieties. Subsequently, endogenous hormone levels were assessed of the two varieties, along with the number of internodes and internode length. The results suggested that the persimmon could be used as a valuable and powerful natural candidate for providing information on the functional role of dwarfing.

## 1. Introduction

The persimmon cultivar “Nantongxiaofangshi” (*Diospyros kaki* Thunb.), characterized by producing performance, is a cultivar suitable for dwarfing and high-density planting due to its weak apical dominance and lack of an obvious trunk. In contrast to “Dafangshi,” “Nantongxiaofangshi” is significantly taller with greater tree height, tree spread, stem diameter, shoot length, and internode length [1]. As one of the rare dwarf persimmon varieties, “Nantongxiaofangshi” has important value for the breeding of dwarf persimmon.

Gibberellin (GA) is a plant hormone that plays an important role in the development of stems and leaves. GA is involved in the regulation of many different growth and development processes, including seed germination, stem and leaf elongation, hypocotyl elongation, flower formation, and fruit development [2]. GA metabolism is associated with dwarfing, yet little research has been conducted on dwarfing in persimmon. GA-related dwarfing in higher plants can be classified into two types: a responsive mutant that is associated with gibberellin signaling and a synthetic dwarf mutant that is associated with the gibberellin anabolic pathway. The synthetic dwarf mutant is principally caused by a GA deficiency due to abnormalities in GA synthetase or other GA metabolic enzymes. Synthetic dwarf mutants can be restored by the application of exogenous GA. In responsive mutants, the growth of such mutants is independent of biologically active GA, or the biologically active GA required for growth is much lower than the normal concentration, which has been identified in several species [3].

The GA biosynthesis pathway in higher plants is controlled by three main types of enzymes, which include *endo*-kaurene oxidase (KO), *ent*-kaurenoic acid oxidase (KAO), GA20ox, GA3ox, and GA2ox. Among them, GA20ox is rate limiting and thus regulates the synthesis of GA [4–6]. The biochemical function of the enzymes involved in GA synthesis has been well characterized in several plant species [7, 8]. In regard to GA signal transduction, DELLA proteins play a key role in the GA signaling pathway [9, 10]. The DELLA family of plant proteins are a large family of proteins that have the ability to inhibit GA-regulated plant growth and development [11]. Five genes in *Arabidopsis* have been reported to affect the GA signaling pathway. Mutations in GAI and RGA affect normal GA response resulting in a dwarf phenotype. The deletions in the N-terminal region of RGA alteration were affected by a deletion of 17 amino acids [12, 13]. GAI and RGA are both members of the DELLA family and play an essential role in seed germination and plant morphogenesis in *Arabidopsis*, rice, and *Brassica* [14–16]. Recently, a strawberry DELLA gene, *FveRGA1*, was suggested to play a role in the formation of strawberry runners [17]. *SlDELLA* has also been proposed to play a role in the formation of individual leaves in tomato, suggesting that GA may act as a negative regulator of leaflet formation [18]. However, few studies have looked for dwarfing genes in persimmons which have been conducted so far.

Deguchi et al. have reported that a cDNA encoding sorbitol-6-phosphate dehydrogenase (S6PDH) of sorbitol biosynthesis from rosaceae was introduced into the Japanese persimmon (*Diospyros kaki*) to increase the environmental stress tolerance and exhibited dwarfing phenotypes [19]. According to our previous research reported, the “Nantongxiaofangshi” interstock has superior characteristic compared to the nongrafted persimmon trees [20]. Academic researches have mainly conducted on fruit trees, such as apple [21], pear [22], and citrus [23]. However, little research of the dwarfing genes has been investigated from the persimmons up till now.

The expression of these genes was assessed at different phenological stages in “Dafangshi” and “Nantongxiaofangshi” persimmon. In the present study, 22 genes were identified in the persimmon variety, “Nantongxiaofangshi,” by transcriptome sequencing that were associated with the gibberellin synthesis and signaling pathway. Lastly, the expression of the 14 genes in response to the hormone applications was also evaluated in the two varieties.

## 2. Materials and Methods

### 2.1. Organism and Chemicals

In the present study, two-year-old “Nantongxiaofangshi” and “Dafangshi” scions grafted on “Junqianzi” rootstocks were obtained from the Fruit Tree Biotechnology Laboratory, Nanjing, China. The leaves sampled from “Nantongxiaofangshi” were taken at the leaf-expanding stage (2018.4.18), the terminal bud senescence stage (2018.5.10), the flowering stage (2018.5.22), the physiological fruit drop stage (2018.6.11), and the fruit color development stage (2018.9.13) [20]. All chemicals used in the present work were purchased from Beijing Solarbio Science and Technology Company Limited (Beijing, China).

### 2.2. Hormone Treatments and Sampling

The hormone treatments consisted of 100 mg·L^−1^ GA_3_ (GA treatment) or 300 mg·L^−1^ PP_333_ (PP_333_ treatment), with distilled water serving as the control (CK). Leaves of the two persimmon varieties were sprayed and make no precipitation in 24 h after the application. Three plants were used for each treatment. Leaves were collected from both the treated groups and the control group at 0 d, 3 d, 6 d, 12 d, and 24 d after treatment. The samples were immediately immersed in liquid nitrogen and stored at -70°C.

### 2.3. Measurements of Shoot Length and Internode Number after Hormone Treatment

New shoot growth (length) and internode number were recorded beginning on the day of the treatment and at 30 d after the treatment. The measurements were taken on three annual shoots of each of the plants that were similar in the two varieties.

### 2.4. Analysis of Endogenous Hormones after Treatment

Endogenous levels of GA_3_, indole-3-acetic acid (IAA), abscisic acid (ABA), and zeatin nucleoside (ZR) were measured using an enzyme-linked immunosorbent assay (ELISA) method [24].

### 2.5. Bioinformatics and Molecular Experiments

#### 2.5.1. Extraction of Total RNA and cDNA Synthesis

Total RNA was extracted from the leaf samples using a Plant Total RNA Isolation Kit Plus (Foregene, Chengdu, China) according to the manufacturer's instructions. RNA integrity was checked by 1% agarose gel electrophoresis, and RNA concentration and purity were measured using a Nanodrop ND 1000 Spectrophotometer. Samples with 28S/18S ribosomal RNA between 1.5 and 2.0 and an absorbance ratio OD260/280 between 1.9 and 2.1 were used in the subsequent transcriptomic analysis. Approximately 1,000 ng of total RNA was used for cDNA synthesis using a PrimeScript RT Reagent Kit with gDNA Eraser (TaKaRa, Dalian, China). The cDNA was diluted with nuclease-free water prior to conducting RT-qPCR.

#### 2.5.2. Selection of Candidate Genes

Based on the transcriptome sequencing data obtained from “Nantongxiaofangshi” leaves (unpublished data), 22 candidate genes related to GA biosynthesis and signaling were cloned. Based on the obtained sequences, gene-specific primers for use in the RT-qPCR analysis were designed using Beacon Designer 7.0 software. A list of the gene-specific primers used in this study are presented in Table 1.

#### 2.5.3. Reverse Transcription Quantitative PCR (RT-qPCR)

RT-qPCR reactions were performed using a LightCycler 480 II System (Roche Diagnostics, Rotkreuz, Switzerland). The PCR reaction mixture (20 *μ*l total volume) included 10 *μ*l of SYBR Premix Ex Taq II (TaKaRa, Dalian, China), 1.0 *μ*l diluted cDNA (150 ng/*μ*l), 8.6 *μ*l ddH_2_O, and 0.4 *μ*l of each primer (10 *μ*M). The PCR conditions were as follows: an initial denaturation step of 95°C (30 s), followed by 40 cycles of 95°C (5 s) and 60°C (30 s). Each sample was assayed in quadruplicate, and the DkGAPDH gene was used as an internal control. Relative expression was determined using the 2^-△△Ct^ method.

### 2.6. Statistical Analysis

All the data were statistically analyzed by the mean ± S.D. (standard deviation). Significant differences were determined using Duncan's mean separation test (SPSS 16.0 software package, USA).

## 3. Results

### 3.1. Expression Analysis of the Candidate GA-Related Genes at Different Phenological Stages of Persimmon

**Figure 1:**
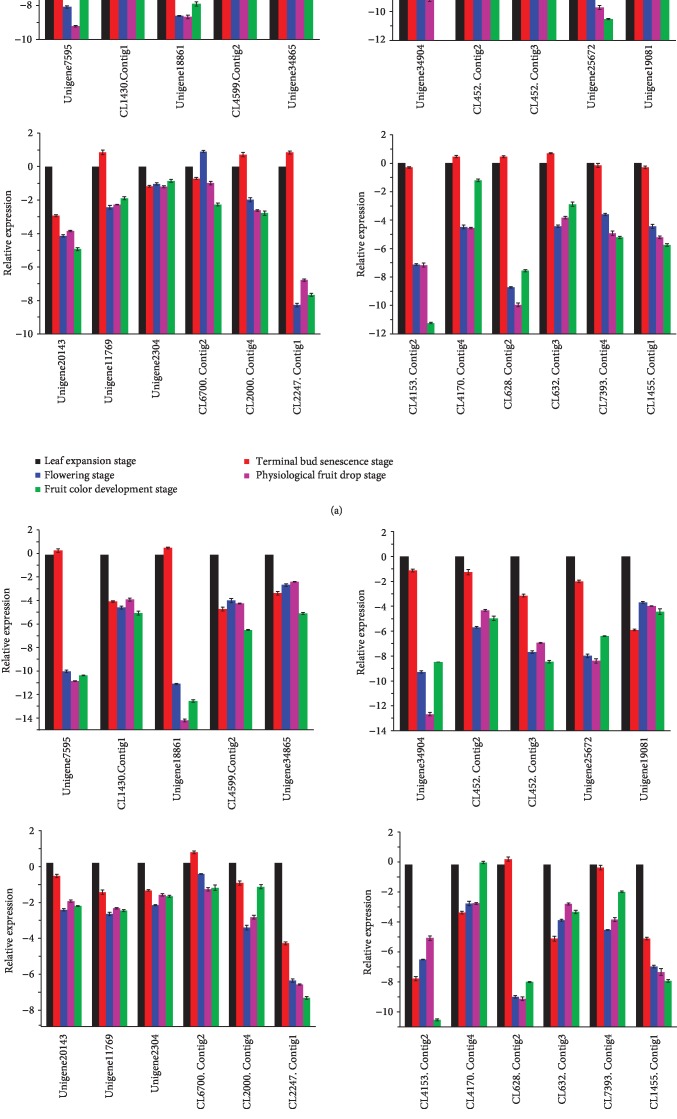
Expression analysis of related genes of Gibberellin biosynthesis pathway in different periods. (a) Dafangshi. (b) Nantongxiaofanghsi.

In this work, the expression level of the 22 candidate genes in “Dafangshi” and “Nantongxiaofangshi” persimmon at different phenological stages was assessed in a preliminary study. The results revealed that 14 of the 22 gibberellin-related genes were differentially expressed at a significant level in the five phenological stages. The expression levels of Unigene7595, Unigene18861, Unigene34904, Unigene11769, CL1455.Contig1, and Unigene2304 were significantly higher in “Dafangshi.” In contrast, the expression level of Unigene20143 and CL628.Contig2 was consistently higher in “Nantongxiaofangshi.” Furthermore, the expression level of CL452.Contig2, CL452.Contig3, Unigene25672, CL4153.Contig2, CL4170.Contig4, and CL7393.Contig4 also exhibited a change in expression specifically at the flowering stage, and an opposite trend was exhibited after flowering (Figure 1).

In detail, the expression levels of these three genes in the whole phenological period were always lower in “Nantongxiaofangshi” than in “Dafangshi,” and the expression levels of these three genes were detected only in “Dafangshi” in the exogenous hormone treatment test. Helliwell et al. pointed out that arabidopsis ga3-1 mutants showed dwarfing characteristics due to lack of *ent*-kaurene oxidase (KO) activity, which was gibberellin-responsive dwarfing, indicating that KO gene expression was tissue specific, and its expression level was higher in growing tissues, but less in mature leaves and resting stems. It was speculated that the low expression of gibberellin synthase-related gene in the “Nantongxiaofangshi” was one of the reasons for its dwarf character.

### 3.2. Effect of Exogenous Hormone Application on Shoot Growth of New Shoots of the Two Varieties of Persimmon

**Figure 2:**
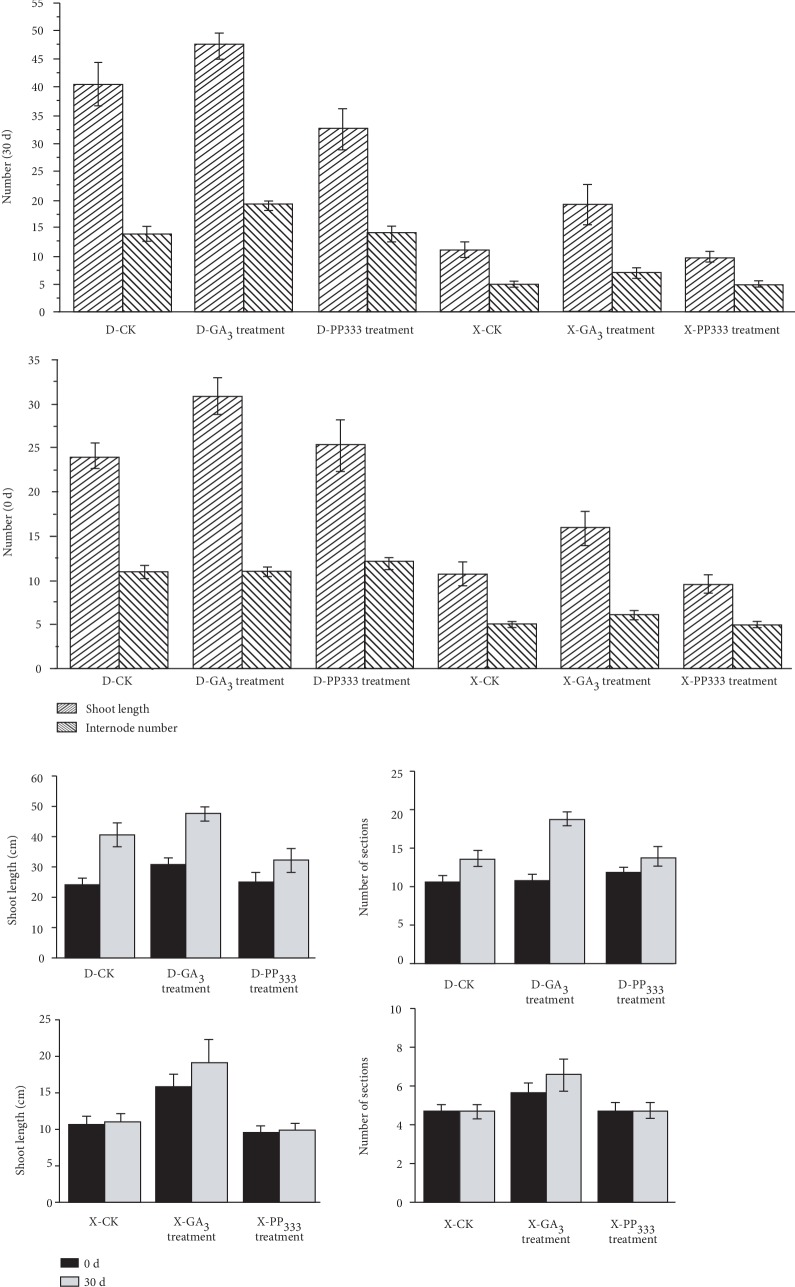
The single 0 d and 30 d for control, GA_3_, and PP_333_ and the effect of GA_3_ (a growth hormone), PP_333_ (a growth inhibitor), and internode production on shoot growth and number sections on two varieties of persimmon.

The length of current-year shoots of “Nantongxiaofangshi” and “Dafangshi” in response to hormone application are presented in Figure 2. Results indicate that the stimulation of shoot growth by exogenous application of GA_3_ was greater in “Nantongxiaofangshi” than in “Dafangshi” and the ability of PAZ to inhibit shoot growth was lower in “Nantongxiaofangshi” than in “Dafangshi.” The number of internodes produced in the two varieties in response to the exogenous application of GA_3_ was greater in both varieties relative to the untreated control, supporting the premise that an application of a sufficient concentration of exogenous GA_3_ treatment can promote shoot growth as determined by the number of internodes produced.

### 3.3. Effect of Exogenous Hormone Applications on Endogenous Hormone Levels: Comparison of Endogenous Hormone Levels in “Nantongxiaofangshi” and “Dafangshi”

**Figure 3:**
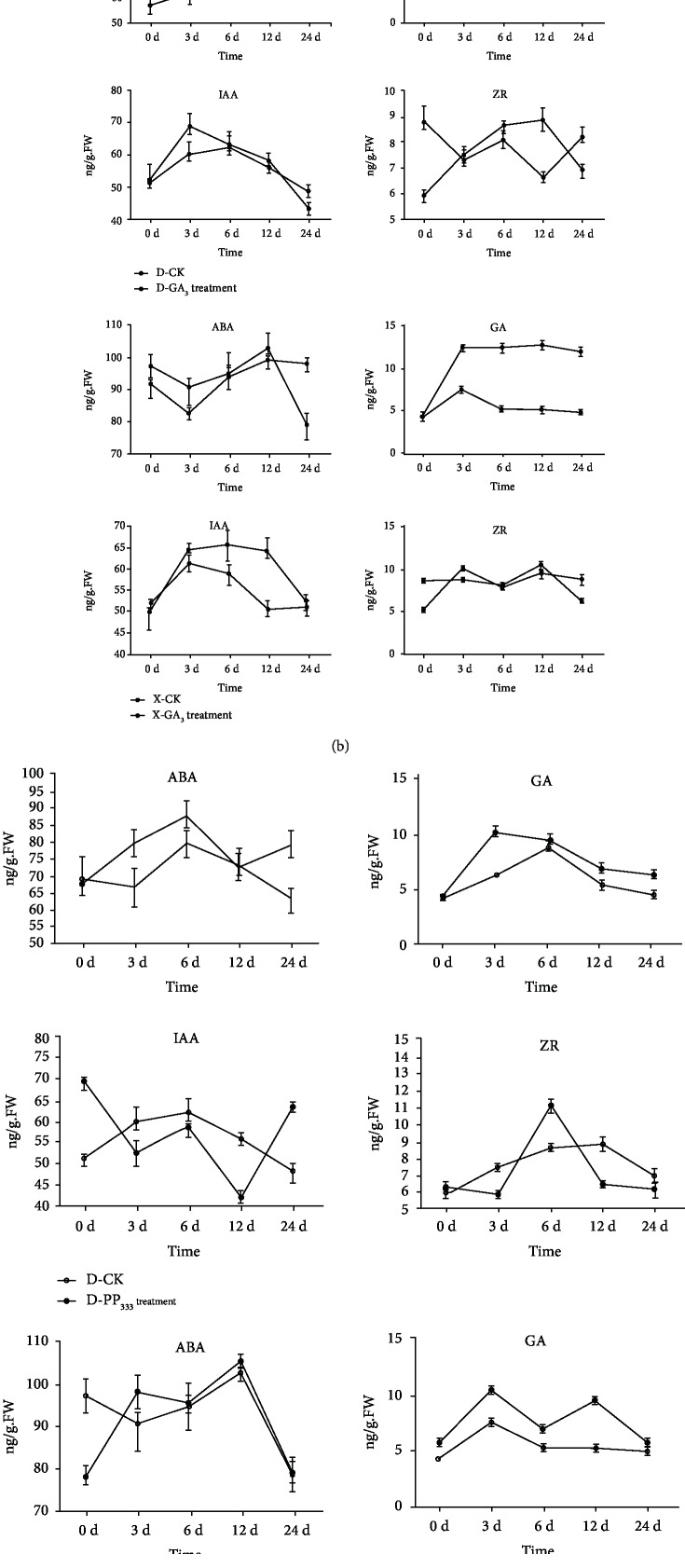
(a) Comparison of endogenous hormone levels in untreated (CK) leaves of two varieties of persimmon. (b) Effect of an exogenous application of gibberellin on endogenous hormone levels in two varieties of persimmon. (c) Effect of an exogenous application of PAZ treatment on endogenous hormone levels in two varieties of persimmon (D: “Dafangshi”; X: “Nantongxiaofangshi”).

Samples were collected from the untreated varieties of persimmon to determine and compare endogenous hormone levels. Results indicated that there was no significant difference between the two varieties in the level of endogenous ZR and IAA. The level of endogenous GA, however, was slightly lower in “Nantongxiaofangshi” than in “Dafangshi,” while the level of endogenous ABA was significantly higher in “Nantongxiaofangshi” than in “Dafangshi” (Figure 3(a)).

These results are similar to those reported in an earlier study [25]. In response to the application of exogenous GA_3_, the endogenous level of GA was consistently higher than that of the untreated control (Figure 3(b)). The concentration of endogenous ABA first increased and then decreased in response to exogenous GA treatment, but was still higher than that of the control after the third day. The level of endogenous ABA decreased in “Nantongxiaofangshi” in response to the exogenous GA treatment. A trend of higher levels of endogenous IAA in the two varieties, relative to the untreated control, was also observed. The response of endogenous levels of ZR was opposite in the two varieties. Overall, the levels of ZR were lower in the GA-treated samples of “Dafangshi,” relative to the untreated control, while differences between the GA-treated and control samples were not obvious in “Nantongxiaofangshi.”

The level of endogenous GA in the two varieties after treatment with exogenous PAZ was always lower than that of the control, while the concentration of endogenous ABA was generally higher, relative to the control. The level of endogenous IAA first exhibited a decline in both varieties in response to the PAZ treatment but then rebounded on the twelfth day. The levels of IAA, however, were generally lower than those of the untreated control. The endogenous level of ZR in both “Dafangshi” and “Nantongxiaofangshi” did not change much, relative to the control; however, ZR levels were slightly higher than the control on the sixth day after treatment (Figure 3(c)).

Specifically, endogenous hormone can cause different variation of persimmon leaves. The content of ABA increases at 6 d after the application for “Dafangshi” and 12 d for “Nantongxiaofangshi,” but it is not sustained with concentrations dropping below that for the controls in the following days. GA increases for both varieties, and this was sustained. Compared with PP_333_, the content of GA increased the most after the management of GA_3_. In addition, IAA also increases for both varieties at 3 d except the treatment of PP_333_ (Dafangshi); this then decreases in GA_3_ but was sustained for PP_333_ until 24 d, where it decreased to control levels. ZR increased in “Nantongxiaofangshi” on 3 d, though after 24 d, both varieties have basically remained approximately degree as before treatment.

### 3.4. Expression Analysis of Candidate Genes in response to Exogenous Application of GA_3_ and PAZ

**Figure 4:**
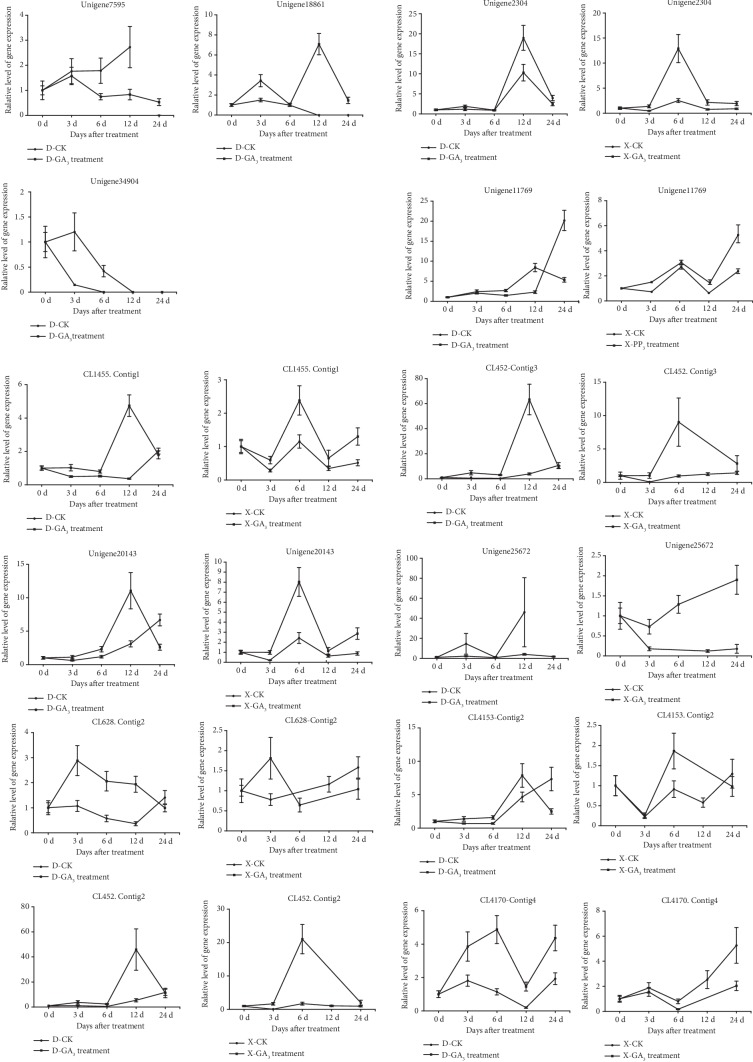
Effect of exogenous GA3 application of gibberellin on the relative expression of gibberellin-related candidate genes in two persimmon varieties.

**Figure 5:**
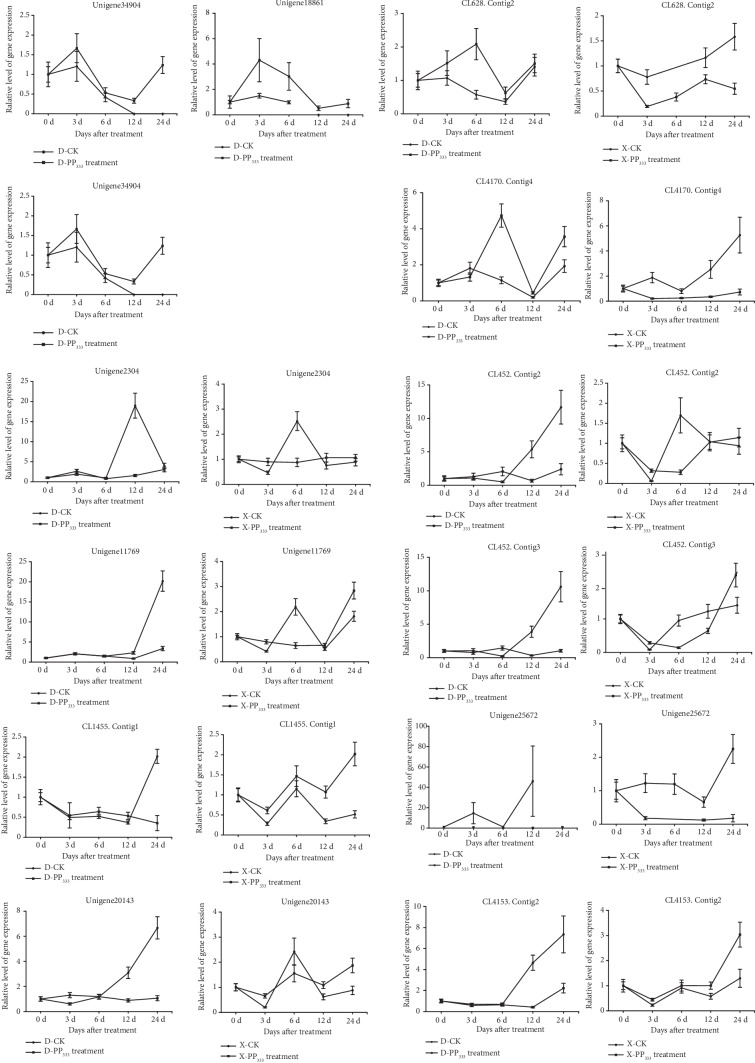
Effect of exogenous application of PAZ on the relative expression of gibberellin-related candidate genes in two varieties of persimmon.

To further determine the difference in expression of the 14 candidate genes identified in this study, their relative expression level was compared in “Nantongxiaofangshi” and “Dafangshi” at a few specific times (0 d, 3 d, 6 d, 12 d, and 24 d) after the exogenous application of GA_3_ and PAZ. Notably, the expression of CL7393.Contig4 was not detected in either variety, so no further analysis was conducted. Three genes, Unigene7595, Unigene18861, and Unigene34904, which are involved in KO synthesis, KAO synthesis, and GA20ox synthesis were only detected in “Dafangshi.” As illustrated in Figure 4, in response to the exogenous application of GA_3_, two genes, Unigene 25672, which is homologous to GA2ox, and CL4170.Contig4, which is homologous to DELLA protein, were significantly differentially expressed in the two varieties in response to GA_3_. In response to the exogenous application of PAZ, the two genes, Unigene 25672 and CL4170.Contig4, were also significantly differentially expressed in the two varieties in response to PAZ. Additionally, the gene CL628.Contig2, which was homologous to DELLA proteins, exhibited significant differences in expression in the two varieties in response to the exogenous application of PAZ (Figure 5).

## 4. Discussions

In the present study, the analysis identified seven candidate genes in “Nantongxiaofangshi,” including three genes encoding KO, KAO, and GA20ox (Unigene7595, Unigene18861, and Unigene34904); one gene encoding GA2ox (Unigene25672); one gene encoding the GA receptor, GID1 (Unigene 2304); and two genes encoding DELLA proteins (CL628.Contig2 and CL4170.Contig4). Among the seven candidate genes, two genes encoding DELLA protein (CL628.Contig2 and CL4170.Contig4) were found to be associated with dwarfing.

Plant growth and development are affected by plant hormone interactions. Gibberellin plays an important role in breaking dormancy and promoting stem elongation. PAZ, as a growth inhibitor, can inhibit GA biosynthesis and cell elongation that reduces internode length in plant stems. The concentration of endogenous GA increased significantly, relative to untreated control, in response to the exogenous application of GA_3_, and endogenous IAA levels also increased slightly. In contrast, increases in endogenous ABA levels were inhibited. In accordance to previous studies, the concentration of endogenous GA and IAA decreased, while the level of endogenous ABA increased subsequent to the exogenous application of PAZ. In previous studies in tobacco [26] and barley [27], the authors indicated that IAA was required for normal GA biosynthesis in stems. Other studies have shown that IAA and DELLA proteins can independently regulate the GA synthesis pathway and promote the accumulation of biologically active GAs [28]. Based on our results, we conclude that endogenous GA and ABA levels are the main factors responsible for the dwarfing traits of “Nantongxiaofangshi.”

GA20ox is a key rate-limiting enzyme for gibberellin synthesis in plants. Sakamoto et al. [24] reported that the level of expression of the GA20ox gene in a pear dwarfing rootstock was related to the height of the plant. Unigene7595, Unigene18861, and Unigene34904 in the present study are homologous to the gibberellin synthase genes, KO, KAO, and GA20ox, respectively. The expression level of these three genes was always lower in “Nantongxiaofangshi” than in “Dafangshi” at all of the examined phenological stages. Notably, the expression of these three genes in response to exogenous application of GA_3_ was only detected in “Dafangshi.” Therefore, we speculate that the low expression level of gibberellin synthase-related genes in “Nantongxiaofangshi” is one of the reasons underlying its dwarfing traits. GA2ox plays a key role in gibberellin inactivation [29, 30]. Several studies have shown that the overexpression of a GA2ox gene promotes excessive metabolic degradation of GA and leads to dwarfing in plants [25, 26, 31]. In the present study, the expression level of three genes homologous to the GA2ox gene (CL452.Contig2, CL452.Contig3, and Unigene25672) changed at the flowering stage in persimmon, suggesting that differences in the expression level of these three genes in “Nantongxiaofangshi” may play a role in its dwarfing trait. Studies in Arabidopsis [30] and rice [26, 31] have shown that there are multiple different GA2ox genes in plant and their expression pattern slightly vary in different tissues.

In the current study, the expression of the gene homologous to the GA receptor GID1 [32] was consistently higher in “Dafangshi” at all of the phenological stages. The expression of this gene was also upregulated in “Nantongxiaofangshi” in response to the exogenous application of GA_3_. This indicates that active GA, as a disguised inducer of GID1, may promote the expression of GID1 in “Nantongxiaofangshi” and promote the degradation of DELLA, and this restores GA-induced plant growth responses. DELLA proteins play a negative regulatory role in GA signal transduction and thus repress plant growth and development. Two GAI genes (Unigene20143 and Unigene11769), homologous to DELLA protein, were upregulated in both varieties in response to the exogenous application of GA, while they were both inhibited in both varieties in response to the application of PAZ. However, CL628.Contig2 and CL4170.Contig4 exhibited a completely different expression response in “Dafangshi” and “Nantongxiaofangshi”; this suggests that CL628.Contig2 and CL4170.Contig4 genes which belong to DELLA protein are associated with dwarfing. Branch orientations in “Nantongxiaofangshi” tend to be horizontal. Previous studies have reported that WEEP plays a role in regulating branch orientation and that silencing WEEP in Prunus domestica (plum) resulted in more horizontal shoot growth relative to standard trees where the orientation of branches is more upright [33, 34]. TAC1 (tiller angle control 1) has also been reported to promote the horizontal growth of branches in Prunus persica (peach) trees, and the attac1 mutant in Arabidopsis exhibits greater upright, vertical branch growth angles [35, 36]. The GA signal transduction pathway is a complex network that is regulated by many factors, such as light signals, hormones, and the environment [37]. In addition, research shows that multiple DELLAs operate synergistically in a plant [38].

## 5. Conclusion

Based on the results of the transcriptome and bioinformatics analysis, we randomly selected genes from the “Nantongxiaofangshi” in 22 gibberellin signal transduction pathways and metabolism-related genes and analyzed the difference from “Dafangshi.” In “Nantongxiaofangshi,” different phenological phases of expressing features, so as screening out the differences associated with gibberellin way of two varieties, have 14 genes. At the same time, in order to explore the dwarf varieties of the “Nantongxiaofangshi” and the dwarf phenotype regulation mechanism of the formation in the growing season of “Dafangshi” and “Nantongxiaofangshi” GA_3_ and PP_333_ foliar spraying processing and determine the content of endogenous hormone after processing and the comparison between their annual side length and number of internode, we analyzed 14 or more genes in the two varieties of the expression of exogenous hormone response differences and ultimately determined the 13 differentially expressed genes. These included 3 genes (Unigene7595, Unigene18861, and Unigene34904), respectively, from KO, KAO, and GA20ox; 3 genes (cl452.contig2, cl452.contig3, and Unigene25672) from GA2ox; 6 genes (Unigene2304) from GA receptor GID1; and 6 genes (Unigene20143 and Unigene11769) from DELLA protein (CL1455.Contig1, CL628.Contig2, CL4153.Contig2 and cl4170.contig4).

Based on previous reports in the literature and the results obtained in the present study, we speculate that CL628.Contig2 and CL4170.Contig4, two genes homologous to DELLA proteins, have involved in dwarfing in persimmon through their regulation of GA signal transduction and its complications.

## Figures and Tables

**Table 1:** Gene-specific primers for the gibberellin-related genes examined in this study.

Gene ID	Molecular function	Primer sequence (5′–3′)	Length (bp)
Unigene7595	*ent*-Kaurene oxidase	CTTGGAACCACCTTGTCTA GGACTTCATCAATGCTTCTC	183
CL1430.Contig1	*ent*-Kaurene synthase	GAGAGGTGGGTTGTAGATAA AGCATCAGATAGTTCAGGAG	107
Unigene18861	*ent*-Kaurenoic acid oxidase	GGAGAGTGAGCAAGTGAT GTGTTGGAGGACTGTTCT	123
CL4599.Contig2	Gibberellin 20-oxidase	CTGGCTTTCTTTCTCTGTC CGCTCTGTAATGCTTCTG	138
Unigene34865	Gibberellin 20-oxidase	TGCCCGAAGAAGGATAAG TGTTGAAGCCAGTAGGAG	164
Unigene34904	Gibberellin 20-oxidase	GCCACTCGTTATCTTGTC TGGAACCGTCATAGTCTC	146
CL452.Contig2	Gibberellin 2-oxidase	AGTTCGGGTTCTTCAAGG GGTGGTTAAGAGGAGATACT	191
CL452.Contig3	Gibberellin 2-oxidase	CCACGACTGTTCCTGATA CTTCCGCCATTAACTCCA	106
Unigene25672	Gibberellin 2-oxidase	CAACCTCTTCAAGACAGATG CCTACGACTGATGACTATGT	109
Unigene19081	F-box protein GID2	GCCATAGGAGCAAGAATTAC GCAATGCCACTCAGAATAC	133
Unigene20143	DELLA protein GAI	CGTCAATGCTGGATCTTC CTTCACCGCCAATAACAC	113
Unigene11769	DELLA protein GAI	ATGCTACTGGCTGTCTTC CGAGTTGGCTAGAGTTGA	157
CL2000.Contig4	DELLA protein	AGTTTGGAAGCAGTAGGG GCATCAGAGGTAGGTTCA	157
CL2247.Contig1	DELLA protein	GTAATGGGAGCGTGAGAT TGAGCAGAAGAACAGAGTC	126
CL4153.Contig2	DELLA protein	CCTTCCATTCCAACTTCTG CTTGGTGCTGGTTGATTAG	127
CL4170.Contig4	DELLA protein	CTTCGTTGCTCAGACTTC GGATACTTCTCGCCAATAAC	198
CL628.Contig2	DELLA protein	GTGCCTGACAATAATGGTAG TGAGAGGTATGACTGAGAAC	102
CL632.Contig3	DELLA protein	CATCATACTGCTGGTCCTA GTCTCATCCTATCCTCATCA	116
CL7393.Contig4	DELLA protein	CCGATTCACAACTTCAGTAG GAGCGTGTAACAGAGGAT	125
CL1455.Contig1	DELLA protein	CGCTTCTTACTTCCTCCA CCATTCTTCTCGTTGACTC	101
Unigene2304	Gibberellin receptor GID1	ATCGGCAGAAGGAGTTAG TCACGGCATCATATAGGG	132
CL6700.Contig4	Gibberellin receptor GID1	GCCTACTGTGAGACATTGA AGTCGGGATTGAAGAGATG	100

## Data Availability

The data used to support the findings of this study are available from the corresponding author upon request.

## Abbreviations

ABA: Abscisic acid
GA: Gibberellin
IAA: Indole-3-acetic acid
KAO: *ent*-Kaurenoic acid oxidase
KO: *endo*-Kaurene oxidase
ZR: Zeatin nucleoside.
